# The Ape That Lived to Tell the Tale. The Evolution of the Art of Storytelling and Its Relationship to Mental Time Travel and Theory of Mind

**DOI:** 10.3389/fpsyg.2021.755783

**Published:** 2021-10-22

**Authors:** Elias Garcia-Pelegrin, Clive Wilkins, Nicola S. Clayton

**Affiliations:** Department of Psychology, University of Cambridge, Cambridge, United Kingdom

**Keywords:** art, evolution, theory of mind, mental time travel, stories

## Abstract

Engaging in the art of creating and telling stories is a defining behaviour of humankind. Humans have been sharing stories with each other, with and without words, since the dawn of recorded history, but the cognitive foundations of the behaviour can be traced deeper into our past. The emergence of stories can be strongly linked to Mental Time Travel (the ability to recall the past and imagine the future) and plays a key role in our ability to communicate past, present and future scenarios with other individuals, within and beyond our lifetimes. Stories are products engraved within the concept of time, constructed to elucidate the past experiences of the self, but designed with the future in mind, thus imparting lessons of such experiences to the receiver. By being privy to the experiences of others, humans can imagine themselves in a similar position to the protagonist of the story, thus mentally learning from an experience they might have never encountered other than in the mind's eye. Evolutionary Psychology investigates how the engagement in artistic endeavours by our ancestors in the Pleistocene granted them an advantage when confronted with obstacles that challenged their survival or reproductive fitness and questions whether art is an adaptation of the human mind or a spandrel of other cognitive adaptations. However, little attention has been placed on the cognitive abilities that might have been imperative for the development of art. Here, we examine the relationship between art, storytelling, Mental Time Travel and Theory of Mind (i.e., the ability to attribute mental states to others). We suggest that Mental Time Travel played a key role in the development of storytelling because through stories, humans can fundamentally transcend their present condition, by being able to imagine different times, separate realities, and place themselves and others anywhere within the time space continuum. We argue that the development of a Theory of Mind also sparked storytelling practises in humans as a method of diffusing the past experiences of the self to others whilst enabling the receiver to dissociate between the past experiences of others and their own, and to understand them as lessons for a possible future. We propose that when artistic products rely on storytelling in form and function, they ought to be considered separate from other forms of art whose appreciation capitalise on our aesthetic preferences.

## Stories of the Self and Their Evolutionary Space and Time

“*How often do we tell our own life story? How often do we adjust, embellish, make sly cuts? And the longer life goes on, the fewer are those around to challenge our account, to remind us that our life is not our life, merely the story we have told about our life. Told to others, but—mainly—to ourselves.”* (Barnes, 2011, p. 104).

Stories and storytelling are ubiquitous parts of our entire existence as a species, and a fundamental feature of our bid to develop our future selves. Every day we tell ourselves and others stories that are detailed with our experiences, our hopes and our future plans. We have a compulsion to solve the riddles implied within the stories we tell ourselves. Story telling is a primary driver in our need to understand our place within the universe and where we may be travelling next. Autobiographical stories describe us travelling the time- space continuum, placing our past self as the protagonist of a story, mentally describing the challenges encountered where and when, and how these were negotiated to achieve a desired future outcome. However, storytelling does not happen in a vacuum: by listening to the stories that others tell us, we are able to gain insight into the challenges that others had, and how these were, or failed to be, surmounted thereby enriching our own mental stories with possible ways of managing our own challenges. The ability to tell stories, be that to others or to ourselves, appears epicentral to both our mental and social lives, as it allows us to plan our future with our past, and other's past, in mind. However, little is known regarding the pressures that selected for this intricate and fundamental exercise, the mental capacities that allowed for it, and how and why these evolved.

Here we will explore these questions by dissecting the art of storytelling into the imperative cognitive abilities for its production, and by scrutinising its adaptive value for both the individual and the group. However, to do so, one must consider, first and foremost, the space and evolutionary time frame by which the behaviour and corresponding cognition evolved. The birth of agriculture emerged but 10k years ago (Cauvin, 2000), and the dawn of recorded history arguably only occurred 5k years ago (Kramer, 1981), and whilst undeniably the human ecosystem has massively changed thanks to these hallmarks, these are, in an evolutionary timescale, minuscule timeframes which seem unlikely to exert selective pressure on the evolutionary development of cognition in a given species. When considering how and why a human behaviour such as the art of storytelling evolved, and its adaptive value, one must not look for fitness in current times, as today's experiences and interactions with the world might be misleading. Evolutionary Psychologists argue that the world is full of examples of processes that evolved to guarantee our survival or reproductive success in the environment of evolutionary adaptiveness [EEA (Bowlby, 1998)], but now, given the rapid environmental changes of the human condition, can be thought of as maladaptive (Crespi, 2000; Miller and Polack, 2018). Therefore, given the misleading nature of today's environment, it would be flawed to think that one can disentangle the adaptive nature of the mind's features by observing the interaction between current human behaviour and current environmental challenges. If one wants to investigate the evolution of art and the cognition that makes it possible, attention should be paid to how evolving art and storytelling practises would have been adaptive for our ancestors in the Pleistocene era, during the 1.8 million years or more that hominids spent as hunter gatherers.

## The Evolution of Art

From the intricate cave paintings that characterise the Upper Palaeolithic, the sculptures of the Renaissance, the Elizabethan theatre, to the largest selling blockbuster of the 21st century, artistic practise has been an omnipresent characteristic of the *Homo sapiens* (Dissanayake, 2015). The rise of Evolutionary Psychology - a field that considers how Darwinian theories of selection have shaped not only the physiognomy of the human brain but also the construction of the human mind (Barkow et al., 1992) - has provided a pathway to researchers who have tried to theorise whether the qualities of art have granted the artists an advantage in surviving or reproducing (e.g., Tooby and Cosmides, 2001; Miller, 2011). Or whether art is but a spandrel of other cognitive adaptations (Pinker, 2003b). While the field investigating the evolution of artistic behaviours does not lack theories of adaptive origin (for a review see Boyd, 2005), little attention has been placed on the cognitive abilities that drive the behaviour. This is a significant query as, without a proper investigation of its imperative cognition, one cannot paint a full picture of the cognitive landscape in which the behaviour evolved. If one is to propose a theory of origin for art, understanding the mental abilities intrinsic in making it possible, and when and why these developed in humans, should be a priority. Dissecting the mental abilities imperative for the employment of a behaviour can help us disentangle the specific qualities of it, thus elucidating the possible proximal problems that could have given rise to the behaviour. However, to fully understand both the cognitive abilities intrinsic in a particular behaviour, and the proximal problems the subject might be solving, one must first strip down the behaviour into its most simplistic form, as otherwise, the inspection would become obfuscated by the surfeit of other factors that might occur as a result of the behaviour, rather than as an originator of it. But how do we strip down art to its most simple essence?

In this paper, our aim is to identify the adaptive need to convey experiences to one another as one of the epicentral differentiators among distinct forms of artistic practises. In order to examine the adaptive need to describe concepts to one another through artistic practise and its evolutionary timeline, we will investigate the relationship between the art of storytelling (i.e., art that entails the transmission and narration of ideas and concepts to the observer), with the cognitive abilities imperative for its production–namely Mental Time Travel i.e., the ability to remember the past and imagine the future (Suddendorf and Corballis, 1997), and Theory of Mind i.e., the ability to attribute mental states to others (Premack and Woodruff, 1978). We argue that the development of both Mental Time Travel and Theory of Mind allowed our species to disseminate past experiences to others for future use, with and without words, giving rise to narrative practises such as the art of storytelling and ritual, and that these practises became intrinsically linked to the survival of both the individual and the group.

## Defining the Art of Storytelling

Art is difficult to define because it encapsulates a group of activities that culminate in distinct artistic products (artefacts). Comparing a sculpture with a concerto, or a painting with a Shakespearian play, seems like a futile exercise as such artistic artefacts do not seem, a priori, to have much in common aside from being categorised under the amalgam term of artistic creation. Perhaps this is because art itself should not be considered necessarily a product, but a behaviour (Dissanayake, 1992). It has been suggested that artistic behaviour has the ability to act as an enhancer of vital activities a society partakes in Dissanayake (1992, 2003, 2015). With the nomenclature “Making special” or “Artifying,” Dissanayake argues that a society that possesses the ability to grant some sort of importance to necessary activities (e.g., hunting or foraging) will be more driven to partake in such behaviours and thus will have a higher survival rate than a society that does not. For example, ritualising hunting by making the hunting tool and hunting protocol special (e.g., decorating the tool and painting hunting scenes) places central significance on the activity, enhancing it and encouraging the society to partake in the behaviour more often or with more precision (Dissanayake, 1992). While Dissanayake's theorem does not take into account the fact that behaviours which are associated to the survival of an organism will, by default, already be special to said organism without the necessity to be highlighted by another behaviour (Boyd, 2005), the importance placed by Dissanayake on the social feature of artistic practise is not misplaced. Edgar Degas (1834–1917) a Parisian artist renowned for his beautiful depictions of ballerinas and pastel artwork once notably remarked:

“*Art is not what you see, but what you make others see.”*

In this quote, the talented artist observed how spectators of art had a different perspective than the artist when observing an artefact, and how authentic artistic skill relied on maximising the transmission of ideas and emotions to the spectators of art. Indeed, the surfeit of different types of artistic products often enjoyed by many have a surplus of incongruences between them, yet they also possess a very inherent commonality. All artistic products, seem, in some way, produced to be observed, understood, or admired by others. Moreover, artists engage in the process of creation to narrate their thoughts, emotions, or even complex ideology regarding the world they find themselves living in, and perhaps it is the finesse with which these ideas are conveyed to the observer of art that might epitomise the skill of the artist. Think, for example of the difference between any of the surfeit of songs encapsulating a tragic love story between two parties, and an epic such as “La Traviata”: while the overall story theme of both examples might not be very different, it is the quality of the method of narration used in the later artefact what characterises it as a masterpiece. This fundamental characteristic of some forms of art, namely that they entrench stories within their foundations, is why we will argue that some art practises may be better considered as a form of sophisticated storytelling between the artist and spectator. It is the narrative embedded within artefacts that we think of as “the story,” and the ability to partake in such levels of communication through art which we call “the art of storytelling.”

## Appreciating the Art of Storytelling

Stories and some art forms appear to be intrinsically linked to each other, making of the artistic products of these practises vessels which artists utilise to reflect how they see the world. However, some artistic products might not be inherently designed to convey a narrative structure; painting and sculpture are prime examples of this as they might not provide the observer with sufficient information to infer a concrete story based on the artefact alone. Indeed, observers' appreciation of an artefact vary both in reference to the artefact being observed, and the type and quality of the observation being employed by the spectator. Appreciating a painting of a landscape, for example, might be based on an adaptation to recognise suitable environments in which to settle, than on social or individual appraises of the sublime (Ruso et al., 2003). Work by Orians and Heerwagen (1992) and Falk and Balling (2010) suggests that affinity for features that can be found in a savanna ecosystem seem to be preferred across cultures. The savannah hypothesis (Orians, 1980, 1986) posits that this is likely to be a result of hominins spending most of their evolutionary history as nomadic hunter-gatherers in the Pleistocene savannah, which might have granted us a sensitivity to landscapes that might provide us with the right number of resources and protection from threats. While modern day humans are, for the most part, not hunter gatherers in the African savannah any longer, this sensitivity to welcoming environments might still be moderating our aesthetic preferences when observing paintings of landscapes. Komar and Melamid (1993, 1997) interviewed a large sample of subjects from nine countries about their predilections (subject and colour schemes, among others) of paintings. Their results showed a strong predilection for natural landscape representations that included water features, an abundance of peaceful animal life and scattered vegetation, while the observers mostly abhorred any kind of abstract representation in the works of art. Similarly that our aesthetic preferences of landscapes appear to be shaped by their relationship with our survival, some of our other perceptions of beauty and sublime might be a product of sexual selection. As Darwin (1871) observed, animals appear to be able to appreciate the plethora of colours and symmetrical features of their sexual counterparts and choose their mate accordingly.

“*This sense has been declared to be peculiar to man. I refer here only to the pleasure given by certain colours, forms, and sounds, and which may fairly be called a sense of the beautiful; with cultivated men such sensations are, however, intimately associated with complex ideas and trains of thought. When we behold a male bird elaborately displaying his graceful plumes or splendid colours before the female, whilst other birds, not thus decorated, make no such display, it is impossible to doubt that she admires the beauty of her male partner. As women everywhere deck themselves with these plumes, the beauty of such ornaments cannot be disputed.”* (Darwin, 1871, p. 94).

Thus, in a similar vein to the way in which female birds of paradise Wahnes's parotia *(Parotia wahnesi)* might find the exaggerated dances of their male counterparts attractive, it is possible that our standards for the movements [including dancing, and performance (Miller, 1999, 2011)], colours [including cosmetics and fashion (Grammer et al., 2003; Power, 2010)], and features that we find beautiful in other humans might be based on our ability to discern the reproductive value of our conspecifics (Bartalesi and Portera, 2015; Buss, 2015).

In contrast to artefacts which rely on aesthetic appreciation alone, artefacts which use the art of storytelling (such as theatre, mime, and dance, to name a few) are reliant on the spectator's ability to infer meaning from the artistic products, which correspondingly is contingent on the artist's skill at communicating such meaning. Consequently, given the artefacts inherently rely on narrative, the observer must engage in a different cognitive process to understand, appreciate, and be affected by it, in comparison to any other art form which does not need to be understood to be appreciated. As we will demonstrate below, in such cases in which the design of art is communicative in nature (i.e., uses the art of storytelling), both Mental Time Travel (Suddendorf and Corballis, 1997) and Theory of Mind (Premack and Woodruff, 1978) are essential cognitive abilities for its appreciation and affect. This might be because, as we will elaborate on in this paper, narrative itself appears to be the product of these cognitive abilities.

### Mental Time Travel

In 1972, Tulving distinguished between two forms of declarative memory: knowing (semantic memory) and remembering (episodic memory) (Tulving, 1972). He argued that the former involves the utilisation of timeless selfless facts about the world. By contrast remembering involves the ability to project the self and time, and to use such information to remember and relive ones' past and to imagine possible future scenarios, which he further elaborated on in his seminal book, Elements of Episodic Memory (Tulving, 1983). He identified two vital phenomenological characteristics of Episodic Memory, autonoesis and chronesthesia. Autonoesis describes a particular self-consciousness that enables us to reflect about our memories and therefore an awareness that we are both the authors and owners of such recollections. Chronesthesia, entails an awareness of the passage of time and our position within it. Taken together, these two types of self- and time-consciousness enable us to position ourselves, in our mind's eye, forwards and backwards in the space-time continuum, remembering past experiences and anticipating possible future scenarios based on our memories of what we think happened in the past and our current state of mind. This Mental Time Travel ability to remember the past and imagine the future has been considered to be epicentral to the development of the human mind (Suddendorf and Corballis, 1997; Wheeler, 2000; Tulving, 2005). Indeed, thanks to this ability one can remember what went well and what did not from an experience and adapt one's behaviour accordingly when faced with similar encounters in the future, thus diminishing the likelihood of repeating similar mistakes and increasing the number of successes (Suddendorf and Busby, 2005). Mental time travel allows us to imagine, in our mind's eye, events that might have never occurred (Clayton and Wilkins, 2018; Wilkins and Clayton, 2019).

In humans, the ability is a core component of the appreciation of any form of art that involve storytelling, as stories are either based on the re-enactment of the episodic memories of the self and others, or otherwise might describe scenarios of impossible events where non-existent entities that ought to be imagined are epicentral characters. Imagination grants us the ability to transcend time and space and gains us access to the large array of possibilities (and impossibilities) our universe has to offer. The relationship between imagination and Mental Time Travel has been extensively investigated (Taylor, 2013; Clayton and Wilkins, 2017), and the ability to imagine has been suggested to be an adaptation that evolved to facilitate the prediction of the consequences of our actions (Suddendorf and Busby, 2005; Tulving, 2005). Indeed, the Constructive Episodic Simulation hypothesis (Schacter et al., 2007, 2008, 2012) argues that episodic memory evolved with the future in mind: rather than preserving an accurate record of what happened, the episodic memory system uses information from past experience to simulate a series of future scenarios, which allows us to juxtapose a number of imagined alternatives to predict and plan for those possible eventualities.

### Theory of Mind

The ability to infer and attribute mental states to others has been suggested to be imperative for the evolution of human societies and its impact on our development as a species has been compared to language acquisition and even bipedalism (Baron-Cohen, 1999)^1^. Regarding its impact on art appreciation, the relationship between art and emotion has portrayed artistic creation and appreciation since the ancient times. Indeed Aristotle noted, in his theory of catharsis, how observing tragedy elicited intense emotions of fear and angst (Schaper, 1968). At a neuronal level, observing artistic products seems to trigger neural correlates of emotional processing (Chatterjee, 2004; Nadal et al., 2008; Cupchik et al., 2009), however little is known regarding our emotional responses to art and their underlying neuronal processes (Skov et al., 2018). The fact that art triggers emotional responses to its spectators is a characteristic element of art appreciation and, perhaps, one of the factors as to why artistic creations are so popular in human societies. Theory of Mind is a conduit for empathy, the ability to be affected by the emotional states of others (Völlm et al., 2006; Singer and Tusche, 2014), and Baron-Cohen and Wheelwright (2004) have argued that empathy is moderated by Theory of Mind because in order to be affected by someone else's mental state, one must first be able to cast aside their own mental state and attribute a different one to the other person. Certainly, to empathise and be affected by the events affecting a hypothetical character in a story, the observer needs to identify the mental states and emotions of the characters depicted in contrast to their own. Being affected by such types of art is a keystone of the experience.

Music is an interesting example to explore as it is multifaceted in its method of delivery; the melody of a song may perhaps be appreciated by different systems than the ones used to appreciate the narration imbedded with it. Humans are by no means the only species that create melodious tunes, however there is discord regarding whether human music can be considered homologous to the tunes of the rest of the animal kingdom (Herzog, 1941; Marler, 2000; McDermott, 2008; Kuroyanagi et al., 2019; Mehr et al., 2019). While several other animals are capable of melodic displays, only humans appear to use music to narrate stories to one another. As such, although the superlative appreciation of human music's melodies has been argued to be a product of sexual selection (Miller, 2000; Ravignani, 2018), it is important to highlight that humans also have the capability to, and often do, appreciate musical pieces for the stories they tell. As such, when spectators engage their imagination, emotion, and empathy when understanding the narrative sequences portrayed within the musical piece, they make use of both their Theory of Mind and their Mental Time Travel ability.

In brief, it appears evident that some art forms fundamentally differ from each other, not only in terms of the type of artefact, be it visual or acoustic or both, but also the process of creation used by the artist, and the cognitive mechanisms that the spectator must use to fully appreciate the art piece. Some artefacts appear to be moderated by human's inherent aesthetic preferences, whilst some other ought to be understood to be holistically appreciated. The use of storytelling as a method of concept diffusion inherent in some artforms may make for a valid differentiator of artistic products, as it appears to rely on specific cognitive processes, namely Mental Time Travel and Theory of Mind, for its interpretation and appreciation. As such, we suggest that when art forms rely on storytelling, they ought to be considered separately from other forms of art which only capitalise on aesthetic preference. This is an important differentiation because, if distinct artistic products differ in both their evolutionary history and the underlying mechanism for its production or appreciation, they might also differ in their adaptive value.

## Evolving the Art of Storytelling

The homo lineage spent a considerable amount of time without engaging in the creation of artistic products (Zilhão, 2007; Powell et al., 2009): although *Homo habilis* already partook in primitive tool making (Wynn, 1993), it wasn't until the cultural revolution in the Aurignacian era (40 ka) that our *Homo sapiens* forefathers started engaging in painting, sculpture and decorative tool making (Bar-Yosef, 2002; Teyssandier, 2008; Porr, 2010). It should be noted however that just because *Homo sapiens* started engaging in artistic practises that could fossilise in the Upper Palaeolithic, that does not mean that before that era our ancestors did not partake in any other artistic behaviours that could not fossilise i.e., singing, dancing, dramatic performance (Dutton, 2009; Garfinkel, 2010; Boyd, 2018). Moreover, whilst it was previously thought that the cultural revolution in the Upper Palaeolithic marked a cultural leap between *Homo sapiens* and archaic species such as Neanderthals and Denisovans [with whom *Homo sapiens* intermixed (Green et al., 2010; Reich et al., 2010; Overmann and Coolidge, 2013)], more recently contemporary archaeologists have started moving away from this notion (Bar-Yosef, 2002, 2007). Indeed, the archaeological record suggests that Neanderthals already partook in artistic behaviours such as cave painting (Hoffmann et al., 2018), sophisticated tool making (Douka and Spinapolice, 2012; Borel et al., 2017; Hoffecker, 2018), and that they may have possessed a form of linguistic communication (Dediu and Levinson, 2018; Botha, 2020), thus it is possible that these were not as cognitively and culturally different to their *Homo sapiens* contemporaries as previously thought (Hawcroft and Dennell, 2000; Finlayson, 2019). Some Evolutionary Psychologists have suggested that the ability to share concepts and notions with one another would have most probably developed before the Upper Palaeolithic (Corballis and Suddendorf, 2007; Corballis, 2009, 2013a,b). The climate shift towards cooler temperatures 2.5 million years ago in the Pliocene epoch resulted in the environment of our ancestors changing from a dense foliage scenery to a more open landscape with less hideouts (Foley, 1987; deMenocal, 2004; Lahr, 2010). This, in turn, probably left our ancestors more vulnerable to predatory conflict while simultaneously forcing them to compete with dangerous predators for protein resources. Such pressures likely forced our ancestors to evolve adaptive mechanisms for survival, in this case a cognitive niche (DeVore and Tooby, 1987) which allowed for sophisticated conspecific cooperation, some level of effective communication and planning as a method of collective survival (Corballis, 2013b). *Homo erectus* already partook in a profusion of complex behaviours such as tool making, cooperative hunting, and fire making (Wynn, 1993; Antón, 2003; Chazan, 2017) this complex battery of behaviours required mastery, thus teaching and learning, and somewhat precise communication between individuals, which probably led to more complex patterns of transmission (Dor, 2015, 2017). Amongst his stages for the development of the modern mind in the *Homo sapiens*, Donald (1993, 2013) posits that the autonomic increase in motor control by *Homo erectus* granted them a new tool of expression, through motor communication, gesture, and body language, which revolutionise their ability for social interaction and expression. Donald suggests that *Homo erectus* evolved a mimetic mind which allowed them to re-enact events to one another, such as the diffusion of new stone tool manufacture techniques, social play, and pedagogic interactions with children (Donald, 1993, 2013). Moreover, intricate memory recall abilities would have been imperative for the deployment and maintenance of such behaviours (e.g., to sequentially remember the complex steps of tool crafting or to tend a fire so it does not die halfway during the night). Corballis proposes the emergence of Mental Time Travel abilities during the Pleistocene, as a result of group cohesion, future planning, and conspecific conflict becoming prevalent factors in our ancestor's ecology (Suddendorf and Corballis, 2007; Corballis, 2013a), however more recent electrophysiological studies of hippocampal recordings in rodents (Moser et al., 2015), and behavioural studies in corvids and other animals give rise to the possibility that it developed much earlier.

Perhaps it was because of the inherent importance of Mental Time Travel ability in the development of the modern human mind that many researchers, across a wide range of disciplines from psychology to poetry (Burns, 1786) have assumed that these abilities are uniquely humans (e.g., Suddendorf and Corballis, 1997; Corballis and Suddendorf, 2007). However, Clayton and Dickinson (1998) challenged this claim by showing that a species of corvid, the Californian scrub-jay can recall the what, where and when of specific past events, and integrate these memories flexibly (Clayton et al., 2001, 2003a) to plan for future events (Clayton et al., 2003b, 2005; Correia et al., 2007; Raby et al., 2007). Of course the issue is to whether, and to what extent, the behavioural components of remembering past events to anticipate future ones captures the phenomenological experiences that accompany mental time travel in humans, especially in the absence of agreed behavioural markers of the phenomenological consciousness involved in the projection of the self in time (Griffiths et al., 1999). Subsequent research has shown that a number of other animals share at least some of these abilities, especially the capacity to recall the what, where and when of past events from non-human apes (Osvath and Osvath, 2008; Martin-Ordas et al., 2010) to rodents (Babb and Crystal, 2006), other corvids (Zinkivskay et al., 2009; Cheke and Clayton, 2012; Müller et al., 2017), and cephalopods (Jozet-Alves et al., 2013; Billard et al., 2020; Schnell et al., 2021a,b). Perhaps this is why some key protagonists arguing for the human uniqueness of Mental Time Travel have now changed their minds (see Corballis, 2013a,b; Boeckle et al., 2020). Corvid behaviours are considered hallmarks of mental time travel in non-linguistic animals. Indeed, their ability to produce rich and flexible representations of past events and prepare for specific future events are comparable to that of human children aged 4–5 years of age (see review by Jelbert and Clayton, 2017).

The ability to remember past experiences and imagine future ones grants the organism an advantage when interacting in novel environments. The capacity to plan for possible futures events presents the individual with more control when living in complex ecosystems in which the events around them might be unpredictable. In humans, the ability to communicate imperative past occurrences to kin or even to the general group, would be of general adaptive value for the survival of the individuals within that group. Moreover, the capability to plan for possible futures events, grants the individual with more control when living in complex social ecosystems in which the actions of their conspecifics are out of their control. Altogether, the pressures encountered by our ancestors, the reliance on social cooperation in early humans alongside an uncanny ability to remember the past and ponder possible futures might have sparked narrative between one another, which probably laid the pavestones for storytelling methods of art such as dramatic performance and dance.

Storytelling is frequently thought to largely be a spoken method of information transmission, in which sophisticated language abilities can easily appear to be imperative for. Yet, while the fact that language provides the transmission of a story with accuracy and descriptive richness is undeniably true (McBride, 2014), it does not necessarily imply that storytelling was non-existent prior to the evolution of language (Laland et al., 2016; Clayton and Wilkins, 2017; Boyd, 2018). Stories can be choreographed, mimed, and re-enacted without engaging in language, think for example of ballet and contemporary dance, in which complex concepts are often transmitted only through movement. Notwithstanding non-linguistic forms of art such as mime, dance, and silent films amongst others, storytelling and language are closely connected to each other, both in the fact that language is extensively used for storytelling practises, and their inherent reliance on both Mental Time Travel (Corballis, 2009) and Theory of Mind (Carlson and Moses, 2001; Astington and Baird, 2005; Hughes and Ensor, 2005; Lillard et al., 2013)^2^. Whether language acquisition preceded art creation or vice versa is a topic of much debate. In the late 1960s, the American geneticist John Pfeiffer suggested that language evolved around 40,000 years ago alongside pictorial art to aid the transmission of knowledge between conspecifics (Issac, 1980). Indeed, the engagement of *Homo sapiens* in such complex artistic practises such as cave depictions required mastery and technical ability, and it characterised another important leap in our aptitude for social learning and transmission of skill as early artists refined their talents (Laland et al., 2016). However, other theorists suggest that language might be a universal feature of humans which must have evolved in the African savannah at least 100 ka ago (Pinker, 1995). Certainly, if language only appeared in the Aurignacian era, the fact that aboriginal Australians and Africans possess language abilities even though they diverged from European humans before the “*Upper Palaeolithic revolution”* occurred does not muster (Miller, 2002; McEvoy et al., 2011; Malaspinas et al., 2016). Corballis (2013a) poses an interesting theory on the evolution of language, namely that grammatical language evolved as a result of the human need to communicate past and future episodes (Corballis, 2009). Language allows us to be precise about the information that we want to transmit, to place our stories within the construct of a particular time dimension, and it appears to be specially well-designed to express interpersonal information such as what happened, where, when, why, and to whom (Pinker, 2003a). The ability to place our experiences within a specific time and place poses a very adaptive advantage as it allows us to communicate to others our experiences so they can use it in the future, and likewise to learn from other's experiences without having undergone them ourselves, thus guaranteeing we do not commit similar mistakes at some point. As noted by Corballis (2013b) the adaptiveness of being able to remember the past and anticipate the future is exceptionally amplified when we are able to remember the past that others underwent. As Santayana (1910) eloquently wrote:

“*Those who cannot remember history are condemned to repeat it.”*

Indeed, Santayana's dictum concisely articulates an essential point regarding information attainment. This being that information, while acquired in the present, is mostly used in the future. However, while this is essential at an individual level, at group level only information that it is transmitted will be used. Different members of a group might have distinct experiences, and such specific knowledge, if properly transmitted will provide the group with essential information that would guarantee survival. Unless the entire group has experienced the history in question, communication is vital. Thus, one could easily transform Santayana's dictum into “*A group that cannot* ***communicate*** *history is condemned to repeat it.”*

## Evolving a Theory of Mind

Premack and Woodruff (1978) first coined the term “Theory of Mind” to suggest that chimpanzees could have the ability to infer their conspecifics mental states and adapt their behaviour accordingly. Since then, the nomenclature has been broadly used in many species of non-human animals and has been deemed to be an intrinsic aspect of advanced human socio-cognitive ability (Adolphs, 2001), enabling us to build shared goals, social contracts, and to cohesively cooperate with each other (Baron-Cohen, 1995, 1999; Ermer et al., 2006; Melis and Semmann, 2010). The idea that the complexity of living in social groups has given a pathway to evolve heightened intellectual faculties has permeated psychology for decades (Chance and Mead, 1953). The primate brain doubles in size when you compare it to other similarly sized mammals (Passingham, 1981; Dunbar, 1998; Dunbar and Shultz, 2007), thus it is not implausible that such a difference in brain size might be influenced by another distinctive feature of the family, their heavily charged social environment (Humphrey, 1976). Amongst the most prevalent theories that could explain for the heightened cognitive abilities that apes possess, is the Machiavellian intelligence hypothesis (Whiten and Byrne, 1988b), in which, inspired by the works of the Italian Renaissance diplomat Niccolò Machiavelli, one must be able to understand the politics of the societal structure you live in, but also be able to shorthand them if beneficial. Social pressures such as the competition with conspecifics for resources and sexual partners in primates shaped the need to evolve deceptive techniques and mental state attribution (Whiten and Byrne, 1988a, 1997).

Machiavellian intelligence extends beyond the primate family. The cognitive capabilities of corvids have been suggested to be alike that of the Apes, and a great example of convergent evolution of cognitive capability (Emery and Clayton, 2004). Food-caching corvids live in socially complex ecosystems in which their caches are liable to pilferage from conspecifics (Clayton and Emery, 2007), as such these large brained birds often alter their caching behaviour in reference to who is watching them cache. These cache protection strategies are complex in nature and have been compared to the intricate techniques of misdirection used by magicians to mislead their audience (Garcia-Pelegrin et al., 2020, 2021; Schnell et al., 2021c). For example, jays will deprive the potential pilferers of visual or acoustic information (Dally et al., 2006; Shaw and Clayton, 2013; Legg and Clayton, 2014), and will perform fake caches in very quick motions, so the observer is unable to pinpoint the location of the real cache (Emery and Clayton, 2001). Moreover, some of these protection tactics are dependent on the experience of the cacher, as they will only be used if they have had prior pilfering experience (Emery and Clayton, 2001). Furthermore, male Eurasian jays *(Garrulus glandarius)* are able to attribute the desires states of their partner and actively adjust the type of food they share in reference to their partner's desire state, irrespective of their own desire for a particular food (Ostojić et al., 2013). Being able to disassociate one's own mental states from that of others might pose an advantage for social animals as it grants the mind reader the ability to anticipate others' behaviour. Living in socially charged environments, in which the optimal social strategy might be dependent on the intentions of others, might create the necessity to evolve cognitive adaptations specialised in mental state inference and attribution (Baron-Cohen, 1995).

**Figure 1:**
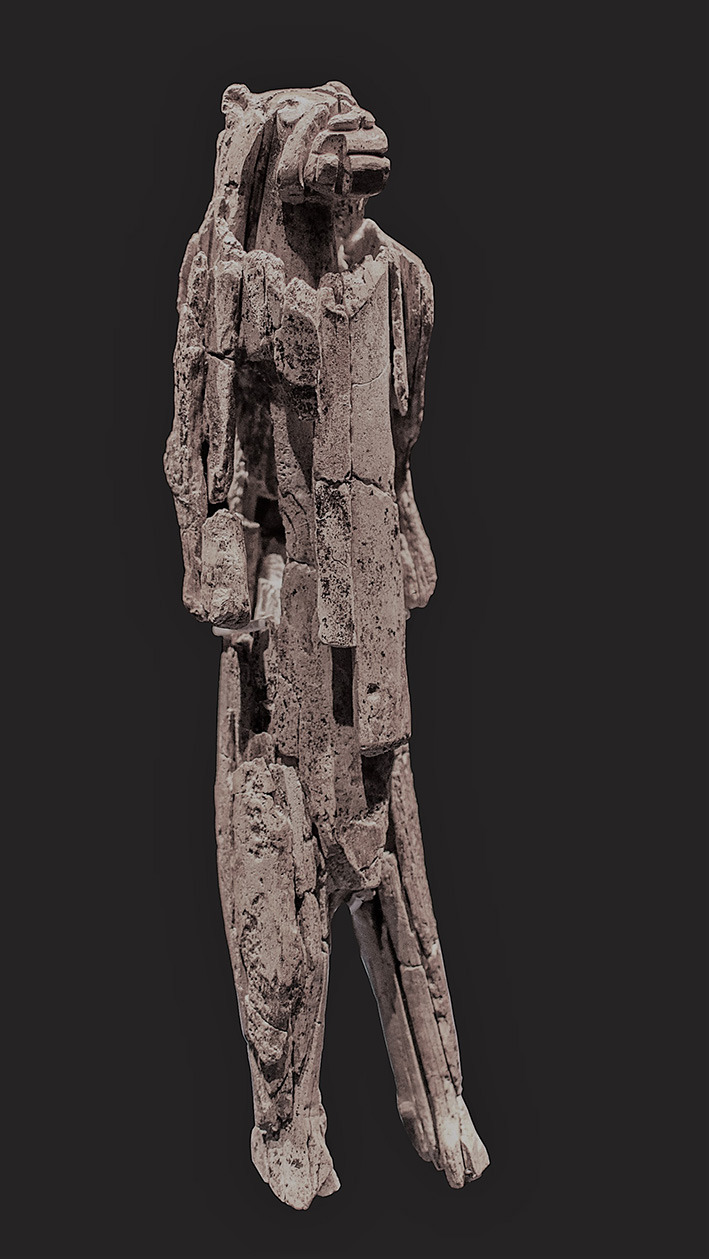
Picture of the Löwenmensch (lion-man) from the Stadel cave in the Hohlenstein, Lonetal made in the Upper Palaeolithic (between 35 and 40k years ago). Picture by Dagmar Hollmann.

The development of Theory of Mind abilities in our ancestors is currently unknown, however, several hypotheses propose different timeframes for when the ability could have originated. On the basis that our closest relatives possess sophisticated mental state inference and attribution abilities, Mithen (1996) suggested that Theory of Mind was possessed by a common ancestor who roamed the earth around 6 million years ago. While non-human apes seem to possess sophisticated mental abilities and can use tactical deception when competing with conspecifics for resources and mates (Whiten and Byrne, 1997), the suggestion that apes possess Theory of Mind akin to humans is a topic of much debate (Kirkpatrick, 2007; Penn and Povinelli, 2007; Heyes, 2017). Field and experimental evidence suggests that non-human apes might possess some degree of Theory of Mind ability, but this does not necessarily mean that they are up to par with the ones that our species possess. As suggested by Baron-Cohen (1999) if current non-human apes had the same Theory of Mind ability, they would also display behaviours that are closely linked to it such as complex communication of shared goals and plans. Thus, suggesting that Theory of Mind most likely evolved after our common ancestor, not before. Interestingly, Baron-Cohen proposes the 33 ka mark as for when we can be certain humans possessed the ability. This is based on the construction of impossible entities such as figures of humans with some animalistic body parts seen in Hohlenstein - Stadel in the south of Germany (Figure 1), which suggests that humans were able to indulge in fiction, a behaviour that involves the artist being able to think about his own thoughts. Baron-Cohen notes how it would be erroneous to conclude that just because our ancestors participated in artistic behaviours such as cave paintings, that they would have, in turn, sophisticated Theory of Mind ability, proposing autistic artists as an example of artists with impaired mental state inference capabilities (Baron-Cohen and Wheelwright, 2004; Pring et al., 2012).

Indeed, through the examination of the material culture left by our hominin ancestors a pattern of the evolution of their socio-cognitive abilities (including the evolution of Theory of Mind) might be drawn out. Cole's (2012) identity model, for example, suggests that the occurrence of increasingly complex artefacts and material culture in the archaeological record can shine a light on the evolution of abstract concepts of identity in ancient hominins. Cole suggests that the ability to communicate meaning to conspecifics through material culture might inherently require the ability to anticipate how the artefact in question will be understood by others, thus implying that the artificer of such material culture understood the difference between their thought process and the one inhered by the observer or user of the artefact in question (Cole, 2015, 2017). Specifically, the appearance of mental templates such as symmetric hand axes in the Lower Palaeolithic (Wynn, 2002) would suggest that early signs of mindreading such as the ability to disassociate their own thoughts, experiences, and ability from other conspecifics might have already been present in hominins as ancient as *Homo erectus*. Archaeological evidence of tool crafting and sharing of symmetric tools between conspecifics templates (Wynn, 2002; Carbonell et al., 2003) might also suggest an ability to alter the personal beliefs of others regarding the self, and the later within group standardisation of tool templates (Shipton, 2013) might suggest a collective identity. Finally, the appearance of artefacts which can be used to individualise a group member such as body ornamentation, which Cole compares to grammatical language, proposes the ability to transmit abstract information about the self.

## Theory of Mind and the Art of Storytelling

**Figure 2:**
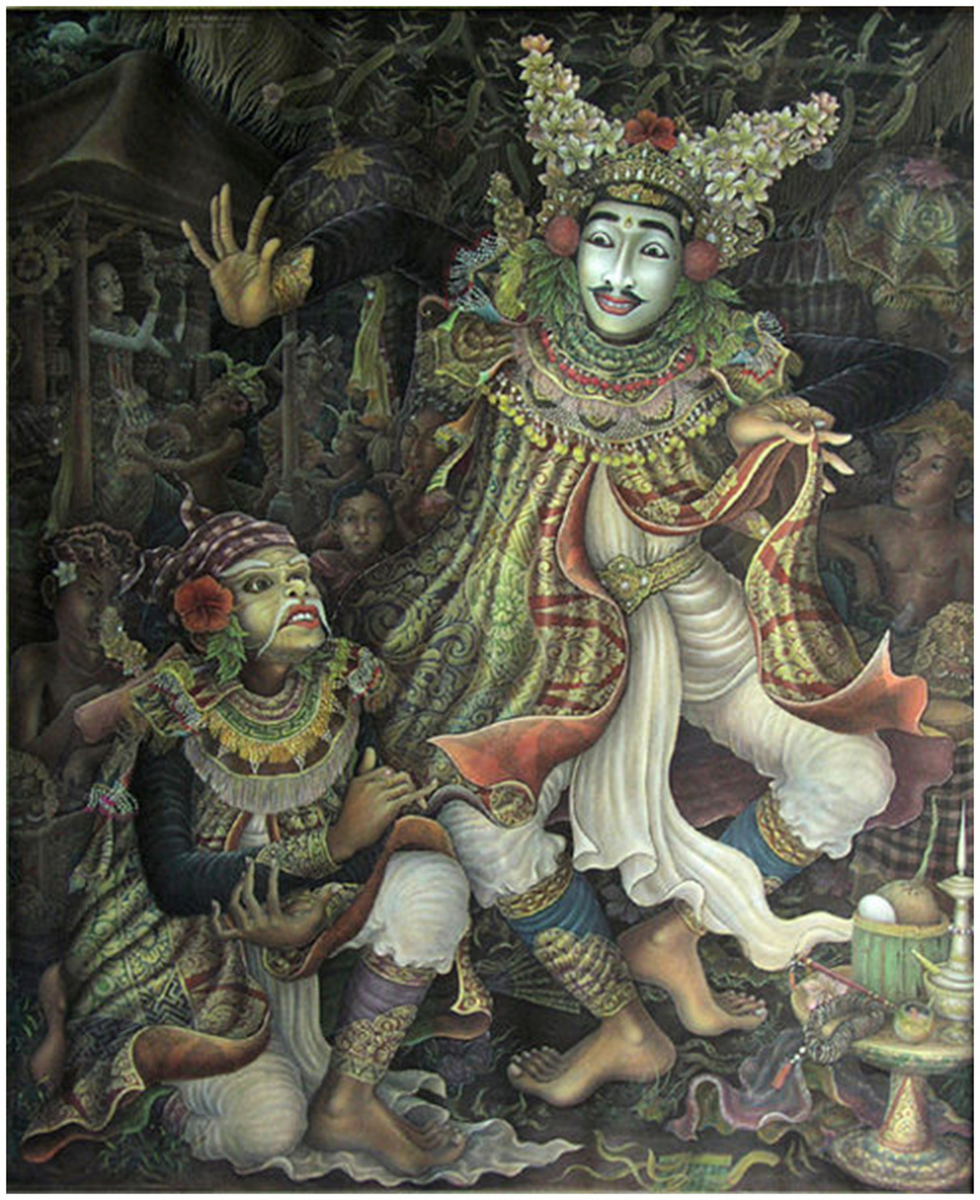
Painting of a Balinese Masked Dancer. Author: Anak Agung Gde Anom Sukawa.

Theory of Mind has often been described as an epicentral feature for effective communication, because, to partake in fruitful communication, all parties involved might need to infer the mental state of one another, be that for transmitting information or for receiving it (Sperber and Wilson, 1995). To communicate a fact or change someone's opinion, one must be able to deduce how the other person's knowledge base differs from their own, and whether the information aimed to be communicated is going to be effective for the desired purpose. Moreover, as new experiences enrich and shape ones' knowledge base, the communicator must also infer how the past experiences of the receiver might have altered the mental state of the receiver since the last time communication took place. Indeed, mental states are not stuck in time, as new information is acquired, or new interactions take place, people often change their views and dispositions towards subjects. Thus, being able to infer such changes is essential for productive communication between individuals. Without Theory of Mind, our communication would be unidirectional information that lacks the art of conversation, the two-way discussion that relies not just on the accurate transmission of information form signaller to receiver, but the reciprocal information in which the receiver becomes the signaller, and the signaller listens to the receiver. For this to be effective both the receiver and the signaller must, in turn, infer the mental state of the communicator to fully interpret the language being used, as meaning and intention are often driven by the mental state of the communicator and the response that is triggered by such information transfer. Evidently storytelling and communication are extremely linked, and diverse methods of storytelling have been used for centuries as a method of information diffusion. Consider Balinese dance (Figure 2), for example, in which both philosophical and devotional epic stories such as the Mahabharata and Ramayana tend to be recreated, practises used as methods of cultural, ethical, and theological diffusion for the population. Similarly, gothic churches have depictions of passages from the bible in their, often colourful, crystal windows, thus ensuring that the 12th century Europeans who had no access to scripture or were unable to read it could still understand the theological dogma.

Alongside communication, Theory of Mind ability is also central to the well-functioning of a society as human relationships are complex and often require access to the mental states of others in order to anticipate their desires and intentions (Baron-Cohen, 1995; Ermer et al., 2006). Storytelling traditions have often been used in rituals for centuries to foster in group cohesion and togetherness. However, while still prevalent in many non-western societies, ritualistic storytelling practises appear to have been forgotten by western societies (Dissanayake, 1992, 2003). This might be due to the 18th-century European philosophy of “Art for art's sake,” that gave birth to modernist and post-modernist art forms, in which the goal of the artist appears to be driven by aesthetics rather than communication (Guerard, 1936; Knieter, 1983). Storytelling traditions are, at least in the non-western artistic practises that remain thriving, a communal effort, in which most members of the community engage in, be that as a storyteller themselves or as a story spectator. The communal aspect of these practises is an imperative feature of the behaviour because creating and partaking in these stories might have a cohesive functionality, by reinforcing the communal beliefs and values through art and tradition, and by promoting trust and cooperation between participants (Sosis and Alcorta, 2003; Sosis, 2004; Whitehouse and Lanman, 2014; Watson-Jones and Legare, 2016). Indeed the development of collective memories [created and delivered through cultural formations such as ritual and monuments (Assmann and Czaplicka, 1995)] not only strengthen the cohesion of the community, but also aid in forming its identity (Manier and Hirst, 2008). Non-western communal storytelling art forms seem, for the most part, to be linked to the diffusion of concepts such as religious belief, philosophical and historical dissemination, and the acknowledgement of important societal occasions such as weddings, coming of age rituals, and funerals. While thematically varying, most non-western ritualistic storytelling ceremonies seem to be displays of their own belief structures, in which they depict their rules and theories (Alcorta and Sosis, 2005). Moreover, these are often performed in periods of disquietude or change for the community and tend to be aimed at actively influencing the group or the events that might be the cause of it (Rappaport and Rappaport, 1999; Dissanayake, 2015).

## Stories and Symbolism

Most types of communication are inherently symbolic, think, for instance, of the utilisation of words or gesturers (Kavanaugh and Engel, 1998; Kavanaugh, 2011). Given the discussion of this paper in which we exemplify the link between art and communication, it is of no surprise that artistic behaviour might follow the same symbolic patterns as other forms of communication. As already mentioned, it appears that stories mainly entail either the retelling of episodic memories (be that the direct memories of the storytellers or the memories of others), or the enactment of fantasy situations, which might vary in their degree of possibility of occurrence but often aim at imparting specific nuggets of information to their audience. The use of symbolism as a delivery concept is an ever-present characteristic of storytelling, which is prevalent in most story types regardless of whether the story is aimed at children or adults. For example, in Dr Seuss's book “How the Grinch Stole Christmas,” (Seuss, 1957) the Grinch steals all the Christmas presents, trees and decorations in Whoville, to later realise that the material elements of Christmas do not encapsulate the Christmas spirit. The message behind this famous children's book (i.e., that a sense of community and belonging is imperative for the well-functioning of a society, and more fulfilling for their inhabitants than amassing wealth or any other materialistic products) is represented by the symbolism behind The Whos, who represent the potential for people to coexist happily, and juxtaposed by The Grinch, which represents bitterness and antisocial behaviour.

Symbolism in storytelling and memory are very closely related to each other. This is because decoding the meaning behind a story's symbolism, often requires self-reflection rather than an *in-situ* appreciation of the semantics that the story underlies. Indeed, appreciating the symbolic representation of stories requires a deep analysis of it, which can only be gained by analysing the complete tale holistically. As such, it is impossible for a spectator of a story, to be able to fully appreciate the symbolic concept underlying the narrative that is currently being experienced, as the spectator does not have access to the full picture of the story. Therefore, the appreciation of symbolic concepts can only be accessed through the afterthoughts of having been told a story, by both recollecting the events that occurred within the tale, and simultaneously disassociating our experience from the memory, thus enabling ourselves to concentrate on the concepts that we missed during such experience and not our experience itself.

Alongside the ability to carefully examine our episodic memories, appreciating the symbolic representations that storytellers use to communicate information is also dependent on Theory of Mind ability (Keskin, 2009; Kavanaugh, 2011), as when observing art, spectators ought to see beyond what is presented to them, and appreciate the artefact for what it might represent, not for what it is. Children under the age of 5 seldom distinguish fantasy and appearances from reality, and do not tend to operate with any other viewpoint in mind than their own (Carlson and Moses, 2001). Thus, as children learn about symbolism and its uses for communication within society, they start seeing past the aesthetical properties of an artefact and start understanding them as communicators of information. The close relationship between Theory of Mind and symbolism is exemplified in the main assessments used to investigate the development of Theory of Mind in children. For example, the false belief task (Wimmer and Perner, 1983), in which the child being tested will be shown information about a situation that others do not possess, and will be asked to infer what others will expect to encounter when interacting in the situation or object (e.g., that inside a cookie jar there are carrots instead of cookies and will be asked to speculate as to what someone else that has not been shown the insides, will think the cookie jar contains). Children that pass this task ought to be able to separate their knowledge of the world, from the knowledge base a stranger to the situation might have, in much the same way as the need to separate knowledge of what I want now from what my future self will want when the future finally arrives (Russell et al., 1999). As such, the child ought to understand that internal mental states have a symbolic relationship with the real world (Perner, 1991; Russell et al., 2010). As children start to develop an understanding of the difference between what it seems and what it actually is, they start gaining an insight into the possibility of the world and the people around them being operated by unseen forces and events that the child has no direct access or influence in. While children usually fully master the telling of their autobiographical stories by the age of 6 (Peterson and McCabe, 1994; McBride, 2014), they start engaging in rudimentary storytelling at the 2-year age mark (Miller and Sperry, 1988) and participate in fantasy storytelling not much later than that (Applebee, 1978). Curiously, similar age-related milestones are found in the development of both Mental Time Travel and Theory of Mind (Nelson, 1993; Fivush et al., 2011; Fernández, 2013; Bauer, 2014), with both abilities having the same cognitive developmental trajectory in young children (Perner, 1988, 1991; Meltzoff, 1999; Russell et al., 2010; Clayton, 2015).

Stories are filled with symbolism, being able to use and infer meaning form these symbolic representations appears central to storytelling and story appreciation, and thus imperative for the development and evolution of a storytelling practise. Mental Time Travel and Theory of Mind are crucial abilities needed to appreciate the symbolic intricacies inherent in the stories we tell, and these two cognitive abilities seem to, in humans, concomitantly develop with storytelling.

## Final Remarks

The stories we tell ourselves are intrinsic to our ability to mentally navigate the space-time continuum by recalling our episodic memories and gaining crucial information from them, whilst imagining possible futures and how to manage them successfully. We feel compelled to do so: we have no other way, it's what we have to do. Storytelling allows us to share our experiences and offer them as lessons for others that might have had different encounters, whilst simultaneously being able to learn from the stories that others share with us.

Evolutionary Psychologists have theorised about the adaptive benefits that engaging in artistic behaviours granted our ancestors, suggesting art and engagement in fiction as an adaptation of the human mind (e.g., Steen and Owens, 2001; Tooby and Cosmides, 2001), a product of sexual selection (Miller, 2002, 2011), or a spandrel of other cognitive adaptations (Pinker, 2003b). However, current theories of art often amalgamate most art forms under one umbrella. Given the inherent reliance on Mental Time Travel and Theory of Mind abilities necessary to both produce and appreciate forms of art reliant on narrative, in contrast to other more aesthetically oriented art forms; we suggest that a clear differentiation ought to be made in reference to whether the art form is used as conduit for storytelling or not. Investigating the central cognitive abilities of a behaviour may grant us a deeper understanding of both the cognitive ability and the behaviour under analysis, as, by doing so, we can gain further insight into the timeframe that the behaviour evolved in, as well as speculating about the proximal problems our ancestors might have encountered, and the way the behaviour under scrutiny impacted such challenges. Mental Time Travel and Theory of Mind are cognitive abilities imperative for the creation and appreciation of performance artistic practises. This is because performance capitalises on our ability to imagine distinct realities and to understand and extract meaning from them. Furthermore, both Mental Time Travel and Theory of Mind abilities are intimately linked with each other, both being vital for language, and a similar developmental trajectory (Perner, 1988, 1991; Meltzoff, 1999; Russell et al., 2010; Clayton, 2015). Certainly, the parallels between art and language both in their use of symbolic representation, inherence in Theory of Mind and Mental Time Travel, and communicative purpose raise the possibility that similar ecological pressures sparked both behaviours.

Storytelling is one of the central characteristics of some forms of art. This is because through artistic practise humans have been communicating concepts to one another at least since the dawn of recorded history, with and without words. Indeed, it is hard to provide adequate evidence as to the purpose of artistic creation such as cave pictorials and figurines in the Pleistocene era, and even harder to do the same with other artistic forms such as dance and storytelling. However, while the lack of fossil evidence makes it harder to assert whether our ancestors partook in in similar practises beyond that time, the stories that our ancestors told to each other live in the cave paintings and figures that they left us, in which some depict dancing men surrounded by musical instruments and body ornaments (Garfinkel, 2010). Certainly, given the inherent communicative nature that performance art seems to possess, one can hypothesise that if our ancestors engaged in storytelling practises such as dancing and performance, they likely possessed the ability to infer meaning from them.

While we undoubtedly use storytelling at a personal level, by recreating our own autobiographical stories, jumping to and from our past, present, and contemplating our future; through storytelling we are also able to share these contemplations with otters, and learn from their own stories in return. Indeed, storytelling and sophisticated communication are sides of the same coin. This is because the art of storytelling not only engages our aesthetic appreciation mechanisms, but also engages our capacity to understand and create meaning, and our need to question the realities that surround us. Mental Time Travel ability seems vital for the evolution of artistic practises as, given the discussion of this paper, most art forms diffuse information of the past for future use. Similarly to Corballis's (2009); Corballis (2013a) propositions that the development of the cognitive ability sparked language proficiencies in our ancestors, it seems likely that it also triggered performance practises given the inherent communicative nature of performance art. Alongside Mental Time Travel, the close relationship between communication and mental state inference makes the evolution of storytelling in art unlikely without the development of a Theory of Mind. Whether engaging in art that uses storytelling is a behavioural adaptation evolved specifically to solve a particular communicative niche or not is yet to be fully theorised and evidenced. However, illustrating how engaging in performance is typically a societal effort that requires the use of sophisticated socio-cognitive abilities such as Theory of Mind, the fact that engaging in performance behaviours would conceivably have some social adaptive quality is hard to deny.

Overall, the investigation of storytelling and its relationship with both Mental Time Travel and Theory of Mind offers crucial insight into our ability to mentally travel backwards and forwards in the space-time continuum, to recognise others' ability to do the same, and to the advantages of doing so, individually and collectively. The evolution of the art of storytelling tells the story of an ape that was able to envision his or her past, imagine their future, and live to tell the tale.

## Author Contributions

EG-P wrote the manuscript. NC supervised the project. All authors contributed equally to the conceptual development of the manuscript and gave feedback on the manuscript.

## Conflict of Interest

The authors declare that the research was conducted in the absence of any commercial or financial relationships that could be construed as a potential conflict of interest.

## Publisher's Note

All claims expressed in this article are solely those of the authors and do not necessarily represent those of their affiliated organizations, or those of the publisher, the editors and the reviewers. Any product that may be evaluated in this article, or claim that may be made by its manufacturer, is not guaranteed or endorsed by the publisher.

## Footnotes

^1^*See the section titled* **“*evolving a Theory of Mind”*** *for more on this*.

^2^*See* ***Theory of*** ***Mind and Storytelling*** *section for more on this*.
